# The potential impact of hereditary hemochromatosis on the heart considering the disease stage and patient age—the role of echocardiography

**DOI:** 10.3389/fcvm.2023.1202961

**Published:** 2023-07-11

**Authors:** Michał Świątczak, Katarzyna Rozwadowska, Katarzyna Sikorska, Krzysztof Młodziński, Agata Świątczak, Grzegorz Raczak, Ludmiła Daniłowicz-Szymanowicz

**Affiliations:** ^1^ Department of Cardiology and Electrotherapy, Faculty of Medicine, Medical University of Gdańsk, Gdańsk, Poland; ^2^ Department of Tropical Medicine and Epidemiology, Faculty of Health Sciences, Medical University of Gdańsk, Gdańsk, Poland; ^3^ Department of Pediatrics, Hematology and Oncology, Faculty of Medicine, Medical University of Gdańsk, Gdańsk, Poland

**Keywords:** hereditary hemochromatosis, speckle-tracking echocardiography, echocardiography, cardiovascular ageing, iron overload (IO)

## Abstract

**Background:**

Hereditary hemochromatosis (HH) is a genetic disease that leads to increased iron accumulation in several organs. Cardiomyocytes are highly susceptible to this damage owing to their high iron uptake, and cardiovascular complications account for 1/3 of the deaths in the natural course of HH. Additionally, excess iron intake and associated oxidative stress may accelerate the aging of the cardiovascular system, regardless of the age of patients with HH. We aimed to investigate the role of standard and speckle-tracking echocardiography (STE) in revealing heart differences in patients with HH considering the disease stage and the patient age.

**Methodology:**

Consecutive patients with HH (*n* = 58) without heart pathologies (except hypertension) and 29 age- and sex-matched healthy individuals underwent echocardiography. Patients were compared according to the time since HH diagnosis (the recently diagnosed HH group [31 patients] with diagnosed HH for less than 6 months and had no more than one venesection; the medium group [11 patients] with diagnosed HH between 6 and 24 months; and the long-lasting group [16 patients] with diagnosed HH for more than 2 years) and the quartile contribution of their age.

**Results:**

Standard echocardiography revealed differences in diastolic parameters between patients with HH and controls, which were the most prominent between healthy and long-lasting HH patients. Regarding systolic function, left ventricular ejection fraction was lower in HH patients, with the most evident differences between the healthy and recently diagnosed HH patients. STE revealed additional differences in systolic parameters, with LV rotation the worst in recently diagnosed patients and its increase in patients with medium and long-lasting HH. Significantly worse peak systolic longitudinal strain values were observed in all patients with HH. Analyses of the results according to the age quartiles of patients with HH revealed that some changes ocurred earlier than expected according to age.

**Conclusions:**

Echocardiography can reveal possible heart damage in HH patients at different stages of the disease and highlight potential features of accelerated myocardial aging in these patients.

## 1. Introduction

Hereditary hemochromatosis (HH) has a genetic etiology in 80% of cases based on HFE-gene mutations, which leads to increased accumulation of iron in bodily tissues resulting in damage to many organs, including the heart (1). Left ventricular (LV) cardiomyopathy was previously responsible for approximately 30% of the deaths among patients with HH (2). The introduction of genetic tests into routine clinical practice for patients with abnormal iron metabolism has enabled the early diagnosis of HH, before an irreversible injury to organs in many cases, decreasing the probability of death.

The mechanisms underlying HH-induced heart damage are not fully understood. Oxidative stress induced by bioactive iron ions, which destroys the tissues of the involved organs, may contribute to this damage (3). Mitochondria are highly sensitive to oxidative stress-related damage, and cardiomyocytes are characterized by a large number of mitochondria, causing the heart to be a particularly vulnerable organ (1). Additionally, iron overload and oxidative stress causes the impaired vascular endothelium vasodilatory function and has a pro-inflammatory effect, with may eventually lead to accelerated aging of the cardiovascular system, with possible effects on the heart, regardless of the actual patients age (4–6). This manifests as an increased ventricular mass attributed to the increased thickness of the myocardium, eventually leading to impaired LV diastolic function (7) and increased left atrial (LA) size (7, 8). These changes can be easily detected using echocardiography (7–9). However, these features are not typical of recently diagnosed patients with HH, who usually have normal parameters on standard echocardiography (10, 11).

However, speckle-tracking echocardiography (STE), a more accurate technique, may reveal changes that indicate deterioration in systolic parameters (such as rotation indices and peak global longitudinal strain) at the early stages of the disease, despite the absence of symptoms and changes in standard echocardiography, presumably due to hereditary character of the disease and heart damage initiated many years before the diagnosis (10, 11). Moreover, as HH leads to changes similar to cardiovascular aging, it remains unknown whether it accelerates cardiovascular aging. Therefore, we aimed to investigate the role of echocardiography in revealing heart differences, considering the disease stage and the patient age.

## 2. Materials and method

### 2.1. Study population

Consecutive patients at different stages of HH [diagnosed based on clinical characteristics, abnormal iron turnover parameters, and the presence of HFE gene mutations (9)] were prospectively enrolled in the study from October 2015 to November 2018. The exclusion criteria were age < 18 years, history of any cardiac diagnosis (apart from hypertension), features of heart damage, and left ventricular ejection fraction (LVEF) < 50%. The control group comprised healthy age- and sex-matched volunteers. All participants underwent echocardiography, and detailed medical histories including duration of HH and administered treatments with laboratory parameters were obtained. The study protocol was approved by the Local Ethics Committee at the Medical University of Gdańsk (NBBN/452/2016), and written informed consent was obtained from all the participants.

The patients were divided into three groups according to the time of diagnosis and initiation of treatment to investigate the influence of HH duration on the heart. The “recently diagnosed” HH group consisted of patients diagnosed with HH for less than 6 months and had no more than one venesection. The “medium” group consisted of patients who had HH diagnosed between 6 and 24 months (2 years). Finally, the “long-lasting” group consisted of patients diagnosed with HH for more than 2 years. All the parameters in these groups were analyzed and compared according to the age distribution obtained during the statistical analyses to investigate the impact of age.

### 2.2. Echocardiography examination

All patients underwent echocardiography at the time of enrollment. Patients were examined in the left lateral decubitus position using a GE VIVID E95 ultrasound system (GE Ultrasound, Horten, Norway) equipped with a phased-array transducer (M5S). Standard echocardiographic parameters were obtained according to the guidelines of the American Society of Echocardiography (ASE) and the European Association of Cardiovascular Imaging (EACVI) recommendations (12, 13). Data acquisition was obtained from the parasternal long- and short-axis views and the three standard apical views. Three consecutive cardiac cycles were recorded during quiet respiration for each view. Grayscale recordings were optimized for LV evaluation at a rate of 50–80 frames/s, and only patients with these parameters were included in the subsequent analyses. All echocardiograms were digitally stored, and further offline analysis was performed using a commercial EchoPAC workstation (v204, GE Healthcare, Horten, Norway).

#### 2.2.1. Two-dimensional speckle-tracking analysis (2D STE)

Three endocardial markers were placed in an end-diastolic frame in the apical four-, two- and three-chamber views to perform a two-dimensional (2D) longitudinal speckle-tracking analysis. The contour of the endocardium was automatically tracked using a software to cover the myocardial thickness of the entire LV wall. Adequate tracking can be verified in real-time and corrected by adjusting the region of interest or manually correcting the contour to ensure optimal tracking. The two-dimensional peak systolic longitudinal strain was analyzed from the apical views and calculated with respect to the strain magnitude at aortic valve closure. The LV apical and basal rotation was quantified by scanning the parasternal basal and apical short-axis planes at the end of the expiratory breath hold. The basal plane was defined as showing the tips of the mitral leaflets, and the apical plane was defined as the level just above the end-systolic LV luminal obliteration. The LV endocardial and epicardial borders of the LV were manually traced. The tracking reliability was visually checked, confirmed, and readjusted when necessary. Counterclockwise rotations viewed from the LV apex were expressed as positive values and clockwise rotations were expressed as negative values. LV twist was defined as the highest net difference in degrees between the apical and basal rotations. LV torsion was defined as the LV twist indexed by the LV diastolic longitudinal length (the distance between the mitral annulus and the apex in end-diastole averaged from the four-, two- and three-chamber apical views). The peak systolic (peak rotation) velocity and early diastolic apical and basal rotation (untwisting) velocities were derived from the rotation rate curves. The untwisting velocity curve was the first negative peak in early diastole, beginning after the peak LV twist and reaching its highest value after mitral valve opening.

### 2.3. Statistics

Continuous data were presented as the medians (25th–75th percentiles), whereas categorical data were expressed as proportions. We performed the Shapiro-Wilk test to determine the normal distribution of our data. Most of the analyzed parameters did not have normal data distributions, even after logarithmic data transformation; therefore, we selected appropriate statistical analysis methods based on non-parametric tests. The significance of differences between the patients with HH and the control group was assessed using the Wilcoxon test (between patients with HH and controls and between groups of patients divided by disease duration and age distribution based on the obtained quartiles). Comparisons between all groups were performed using the Kruskal–Wallis test for continuous variables (with Dunn's post-hoc test for the multiple comparisons with Bonferroni adjustment to determine the significantly different pairs of groups and control for the overall error rate when conducting multiple comparisons) or by the chi-squared test or Fisher's test for categorical variables. Statistical significance was set at *p*-values <0.05 were considered significant. Statistical analyses were performed using R version 4.2.1. (R Core Team, Vienna, Austria).

## 3. Results

### 3.1. Comparisons between the whole HH group and healthy participants

Fifty-eight consecutive patients diagnosed with HH between 1 month to 20 years were enrolled in the study. Forty-one patients had the C282Y/C282Y mutation, 12 had the C282Y/H63D mutation, four had the H63D/H63D mutation, and one had the C282Y/WT mutation. The median patient age was 45 years (range: 31–57 years). Table 1 shows the demographic data, medical history, and laboratory results of all the patients with HH. Twenty-nine age- and sex-matched healthy volunteers comprised the control group.

**Table 1:** The HH patients’ characteristics at the time of the first contact.

	HH All *n* = 58
Age	47 (31–57)
Male sex	66%
Hypertension	38%
DM/IGGT	36%
Months from the time of diagnosis	24 (3–94)
Number of venesections	1 (0–22)
Iron [ug/dl]	205 (169–229)
Ferritin [ng/ml]	581 (274–1,037)
Haemoglobin [mg/dl]	15.1 (14.5–16.1)
TSAT [%]	81 (63–93)
Glucose [mg%]	96 (87–101)
ASPAT [U/L]	28 (21–43)
ALAT [U/L]	40 (26–76)

Data are presented as the medians (25th–75th percentile); TSAT, transferrin saturation; DM, diabetes mellitus; IGGT, impaired gestational glucose tolerance; ALAT, alanine aminotransferase; ASPAT, aspartate aminotransferase.

**Figure 1:**
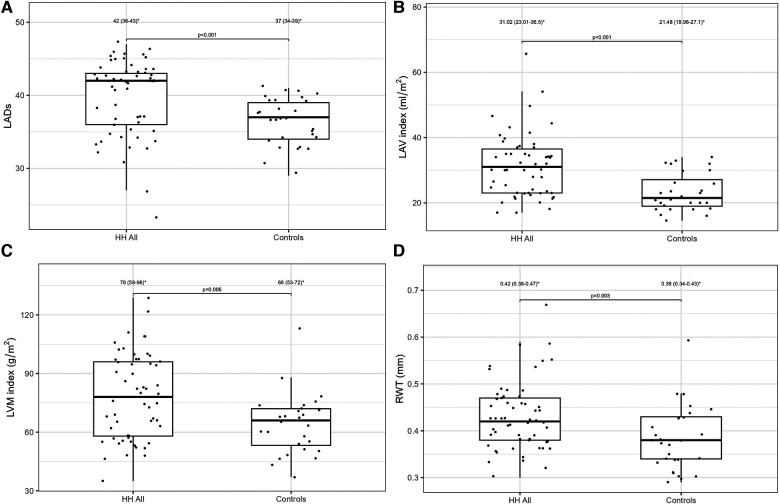
LADs (**A**), LVM index (**B**), LAV index (**C**), and RWT (**D**) values between all HH patients and the control group. The dots show the values obtained by each person analyzed. * Data are presented as medians (25th–75th percentile).

**Figure 2:**
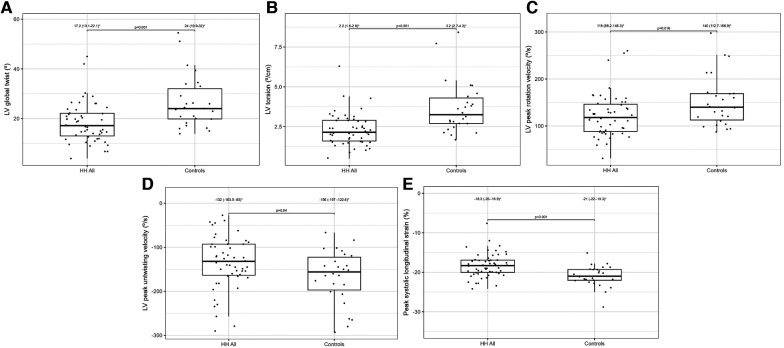
Lv global twist (**A**), LV torsion (**B**), LV peak rotation velocity (**C**), LV peak untwisting velocity (**D**), and peak systolic longitudinal strain (**E**) values between all HH patients and the control group. The dots show the values obtained by each person analyzed. * Data are presented as medians (25th–75th percentile).

Table 2 presents a comparison of the echocardiographic examination between patients with HH and healthy individuals. Some parameters related to diastolic function [LA size (LADs, LAV index), IVS, PW, RWT, and LVM index] were significantly worse in patients with HH than in healthy individuals (Figure 1). The LVEF was within the normal range; however, it was significantly lower in patients with HH. There were no significant differences in the LV and the right ventricle sizes. We found differences in many 2D STE parameters, including those related to diastolic function (LV untwisting rate and velocity), and in many related to systolic function (LV apex rotation, global twist and torsion, and peak systolic longitudinal strain; Figure 2).

**Table 2:** Echocardiographic comparison between HH and healthy persons.

	HH All *n* = 58	Controls *n* = 29	*p*
IVS (mm)	10 (10–12)	9 (7–10)	<0.001
PW (mm)	9 (8–11)	8 (7–9)	0.007
Em (cm/s)	0.10 (0.09–0.13)	0.12 (0.09–0.14)	0.059
E/Em	7.0 (5.6–8.3)	6.7 (5.0–7.5)	0.071
LVEDD (mm)	46 (43–48)	44 (42–47)	0.052
LVESD (mm)	28 (25–30)	28 (26–30)	0.442
LVEF (%)	60 (54–62)	63 (61–65)	0.006
RVID (ms)	27 (24–29)	26 (22–29)	0.169
LV basal rotation (°)	−5.5 (−7.1–−2.8)	−7.1 (−8.4–−4.1)	0.063
LV basal rotation velocity (°/s)	−53.6 (−73.3–−22.9)	−51.5 (−60.3–−25.0)	0.406
LV basal untwisting velocity (°/s)	51.1 (31.5–77.9)	48.5 (40.0–62.3)	0.422
LV apical rotation (°)	11.5 (7.9–17.0)	16.9 (13.8–24.5)	0.011
LV apical rotation velocity (°/s)	82.0 (54.5–105.6)	100.0 (56.0–114.0)	0.095
LV apical untwisting velocity (°/s)	−90.0 (−118.0–−59.5)	−130.5 (−167.7–−107.5)	<0.001

Data are presented as medians (25th–75th percentile). IVS, intraventricular septum (mm); PW, posterior wall; E, early mitral velocity; Em, peak mitral annulus velocity; E/Em, early mitral inflow velocity to peak mitral annulus velocity ratio; LVEDD, left ventricle end-diastolic diameter; LVESD, left ventricle end-systolic diameter; LVEF, left ventricular ejection fraction; RVID, right ventricle internal diameter; LV, left ventricle.

### 3.2. Comparisons between HH patients at the different stages of the disease

Table 3 shows the demographic data, medical histories, and laboratory results of patients with HH at the different stages of the disease. There was an increasing trend in the age and frequency of hypertension in the analyzed HH groups, and the rate of glucose disturbances increased noticeably in the long-lasting HH group. Iron levels were stable between the groups with reduced ferritin concentrations in medium and long-lasting HH patients. TSAT and transaminases levels were similar between groups.

**Table 3:** The HH patient group's characteristics at the time of first contact.

	Recently diagnosed HH *n* = 31	Medium HH *n* = 11	Long-lasting HH *n* = 16
Age	36 (31–54)	44 (30–55)	55 (51–62)
Male sex	20 (65%)	8 (73%)	10 (63%)
Hypertension	7 (23%)	5 (45%)	10 (62%)
DM/IGGT	8 (26%)	3 (27%)	9 (56%)
Months from the time of diagnosis	6 (1–14)	37 (30–52)	152 (121–183)
Number of venesections	0 (0–1)	4 (3–6)	53 (31–76)
Iron [ug/dl]	176 (148–223)	164 (125–178)	165 (134–222)
Ferritin [ng/ml]	422 (272–1,050)	280 (212–430)	190 (66–234)
Haemoglobin [mg/dl]	15.1 (14.3–16.1)	14.2 (13.9–15.4)	15.3 (14–16.1)
TSAT [%]	82 (62–93)	65 (55–83)	72 (68–81)
Glucose [mg%]	94 (87–99)	98 (94–104)	103 (97–105)
ASPAT [U/L]	25 (19–38)	23 (17–35)	24 (21–39)
ALAT [U/L]	33 (23–60)	35 (29–70)	26 (23–44)

Data are presented as the medians (25th–75th percentile); TSAT, transferrin saturation; DM, diabetes mellitus; IGGT, impaired gestational glucose tolerance; ALAT, alanine aminotransferase; ASPAT, aspartate aminotransferase.

**Figure 3:**
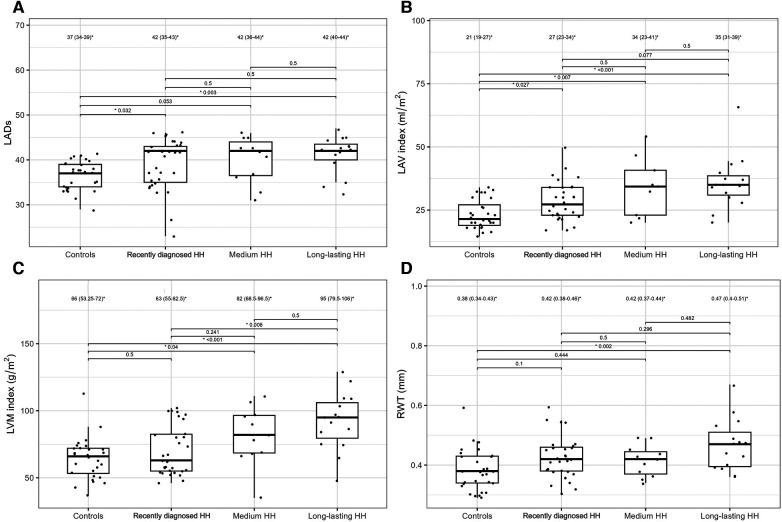
LADs (**A**), LAV index (**B**), LVM index (**C**), and RWT (**D**) values of the controls and HH groups divided according to the stage of the disease. The dots show the values obtained by each person analyzed. The values above the brackets correspond to the *p*-value. * Data are presented as medians (25th–75th percentile).

**Figure 4:**
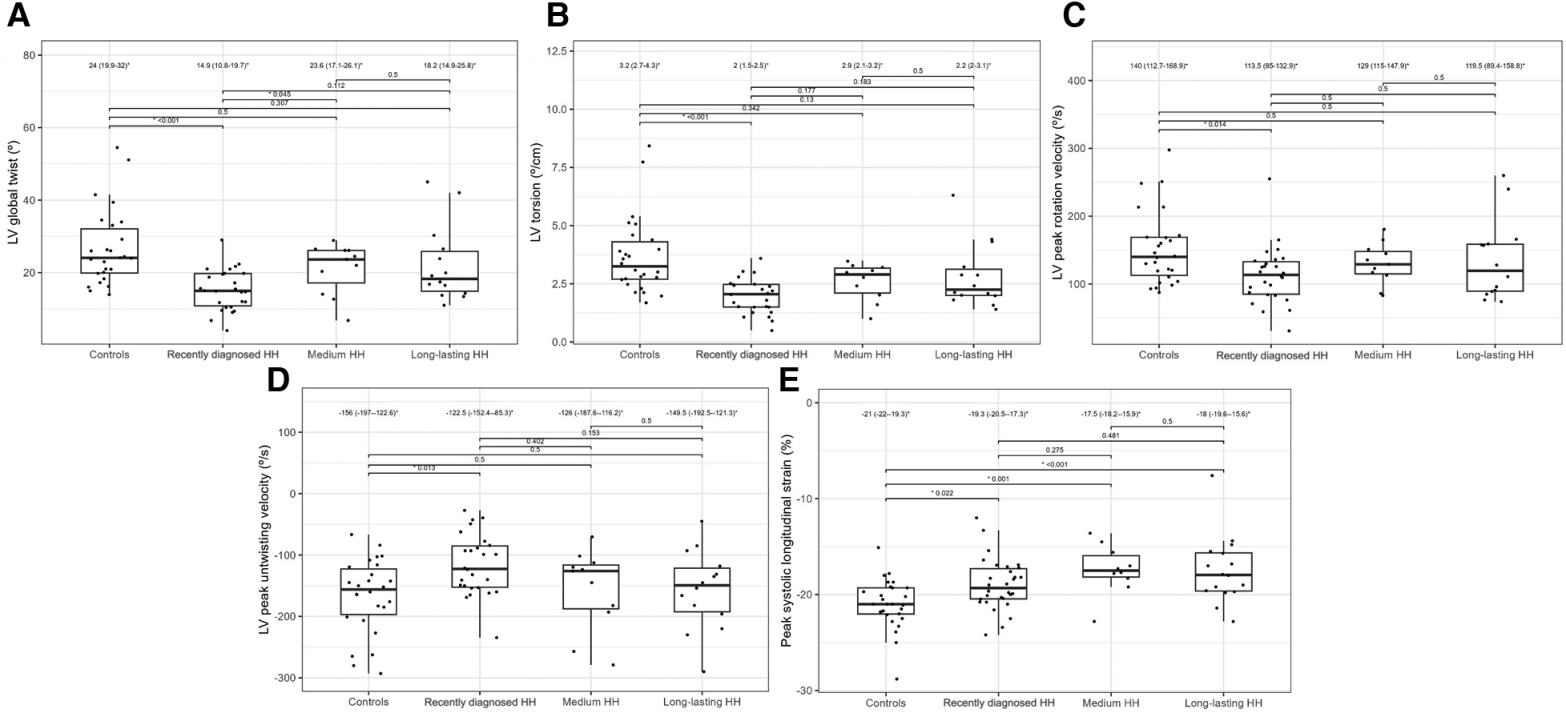
LV global twist (**A**), LV torsion (**B**), LV peak rotation velocity (**C**), LV peak untwisting velocity (**D**), and peak systolic longitudinal strain (**E**) values of the controls and HH groups divided according to the stage of the disease. The dots show the values obtained by each person analyzed. The values above the brackets correspond to the *p*-value. * Data are presented as medians (25th–75th percentile).

Table 4 presents the comparisons of the standard and 2D STE echocardiographic parameters between HH patients in terms of disease stage. Significant differences in LA size were observed between the controls and each HH group but not between the patients with HH from each group (Figure 3). Differences in LV wall thickness, RWT, and LVM index were the most prominent between healthy volunteers and patients with long-lasting HH (Figure 3). Differences in LVEF were the most evident between the healthy volunteers, the recently diagnosed group, and the medium HH group (Table 4). Regarding the 2D STE parameters, the medium group had significantly better rotation indices than the recently diagnosed patients, with no significant differences compared to the long-lasting HH group. Regarding peak systolic longitudinal strain, significantly worse values were noticed in all the HH groups compared to the controls, with no significant changes between the HH groups: −19.3% [−20.5–−17.3] in the recently diagnosed group, −17.5% [−18.2–−15.9] in the medium group, and −18.0% [−19.6–−15.6] in the long-lasting HH group, contrary to −21.0% [−22.0–−19.3] in the control group (Figure 4).

**Table 4:** Comparison of standard and 2D STE echocardiographic parameters between the controls and HH groups.

	Controls *n* = 29	Recently diagnosed HH *n* = 31	Medium HH *n* = 11	Long-lasting HH *n* = 16	*p*
IVS (mm)	9 (7–10)^§,¶^	10 (8–11)^¶^	11 (10–11.5)^	12 (10.5–13)*^,^^	<0.001
PW (mm)	8 (7–9)^¶^	9 (7–10)^¶^	9 (9–10.5)	11 (9.5–12)*^,^^	<0.001
Em (cm/s)	0.12 (0.09–0.14)^¶^	0.11 (0.09–0.15)^¶^	0.13 (0.09–0.13)	0.09 (0.09–0.10)*^,^^	0.030
E/Em	6.7 (5.0–7.5)	6.56 (5.48–7.90)	7.33 (5.90–8.42)	7.70 (6.57–9.30)	0.161
LVEDD (mm)	44 (42–47)	45 (43–48)	48 (44–50)	46 (45–47)	0.235
LVESD (mm)	28 (26–30)	28 (26–29)	30 (27–31)	28 (25–32)	0.538
LVEF (%)	63 (61–65)*§^,^	60 (56–62)^	57 (53–61)^	62 (58–66)	0.009
RVID (ms)	26 (22–29)	25 (24–29)	27 (26–28)	29 (22–29)	0.182
LV basal rotation (°)	−7.1* (−8.38–−4.13)	−3.5 (−5.8–−1.7)^¶^^,^^	−5.8 (−6.6–−3.0)	−6.8 (−9.4–−6.1)*	0.020
LV basal rotation velocity (°/s)	−51.5* (−60.3–−25)	−38.0 (−68.8–−1.0)^¶^^,^^	−53.0 (−67.3–−40.2)	−68.0 (−78–−54.2)*	0.070
LV basal untwisting velocity (°/s)	48.5 (40.0–62.3)	43.8 (25.5–63.1)^¶^	49.0 (25.6–79.4)	75.9 (59.3–79.8)*	0.024
LV apical rotation (°)	16.9 (13.8–24.5)*	10.9 (6.28–14.3)^§^^,^^	17.7 (13–22)*	12.0 (9.28–16.9)	<0.001
LV apical rotation velocity (°/s)	100 (56–114)	69.8 (42.4–99.3)	98.0 (83.7–113)	76.3 (60–126.8)	0.159
LV apical untwisting velocity (°/s)	−130.5 (−167.7–−107.5)*	−76 (−103.6–−56.5)^	−102.7 (−122.7–−73.3)	−111.5 (−130.8–−65.9)	0.003

Data are presented as medians (25th–75th percentyl). IVS, intraventricular septum (mm); PW, posterior wall; E, early mitral velocity; Em, peak mitral annulus velocity; E/Em, early mitral inflow velocity to peak mitral annulus velocity ratio; LVEDD, left ventricle end-diastolic diameter; LVESD, left ventricle end-systolic diameter; LVEF, left ventricular ejection fraction; RVID, right ventricle internal diameter; LV, left ventricle. *p*-value: for differences among all groups with Kruskal–Wallis test for continuous variables or with chi-square test for categorical variables, *p* < 0.05 in posthoc tests for differences with group recently diagnosed (*), medium (^§^), long-lasting (¶) or controls (^).

**Figure 5:**
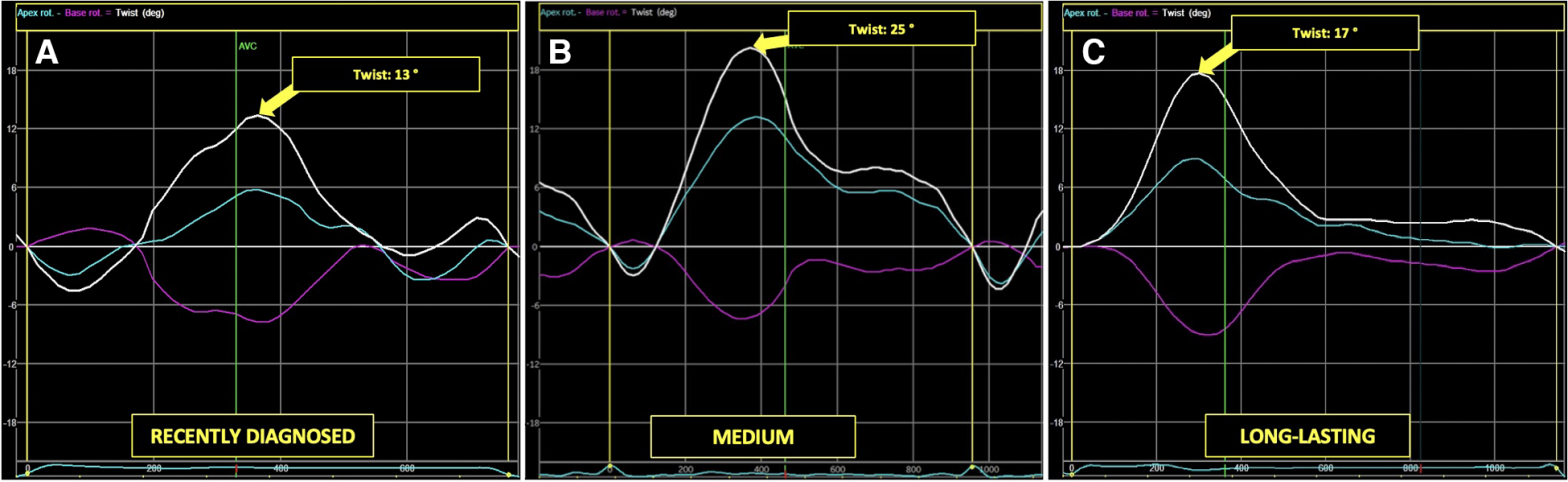
The example of LV apical (blue line), basal (pink line), and peak LV rotation twisting (arrow) curves in HH patients at different stages of the disease: the recently diagnosed (**A**), the medium (**B**), and the long-lasting (**C**).

**Figure 6:**
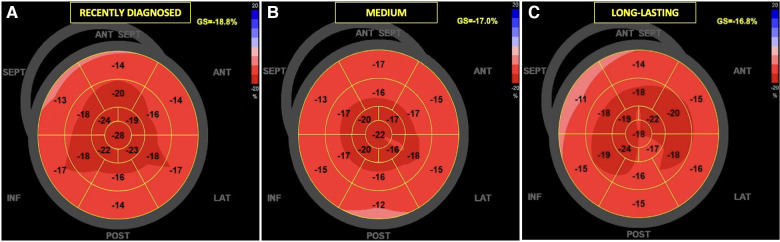
The examples of global longitudinal strain in HH patients at different stages of the disease: the recently diagnosed (**A**), the medium (**B**), and the long-lasting (**C**). In the “bull's-eye” map, all myocardial regional deformations, from basal to middle and apical segments are displayed in a single image.

Figures 5, 6 present three HH patients from each group with graphical demonstration of twist and peak systolic longitudinal strains.

### 3.3. Comparisons between HH patients stratified by age

We divided patients with HH into four groups according to age quartiles based on age distribution to investigate the influence of the age on heart changes: the quartile from minimum to Q25 included patients from 18 to 31 years; Q25–Q50 included patients from 32 to 47 years; Q50–Q75 included patients from 48 to 56 years old; and the fourth quartile (Q75 to maximum) included patients from 57 to 77 years. Table 5 shows the demographic data, medical history, and laboratory results of the patients with HH stratified by age. Among the patients with HH, the incidence of hypertension and diabetes increased with age. The rate of glucose disturbances increased in patients aged 32–47 years. Iron levels were stable between the groups, with reduced ferritin concentrations among patients aged 32–47 years and 57–77 years. The levels of TSAT and transaminases were comparable in both.

**Table 5:** The HH patient group's characteristics at the time of first contact.

	Age 18–31 year *n* = 17	Age 32–47 year *n* = 14	Age 48–56 year *n* = 13	Age 57–77 year *n* = 14
Male sex	77%	79%	69%	36%
Hypertension	12%	14%	62%	71%
DM/IGGT	12%	36%	39%	57%
Months from the time of diagnosis	6 (3–25)	19 (3–36)	85 (24–160)	49 (3–145)
Number of venesections	1 (0–1)	1 (0–6)	27 (1–62)	4 (0–49)
Iron [ug/dl]	170 (155–200)	161 (129.75–199)	171 (151–211)	178 (135–219)
Ferritin [ng/ml]	331 (180–640)	334 (224–784)	279 (217–430)	227 (191–457)
Haemoglobin [mg/dl]	15.8 (14.2–16)	15.7 (14.9–16.6)	14.5 (13.7–15.4)	14.8 (13.8–15.1)
TSAT [%]	71 (56–82)	80 (60–91)	68 (65–77)	85 (69–90)
Glucose [mg%]	87 (85–94)	98 (93–114)	98 (96–103)	98 (97–104)
ASPAT [U/L]	23 (17–33)	38 (20–42)	24 (20–38)	24 (22–26)
ALAT [U/L]	40 (23–66)	45 (29–83)	27 (24–37)	31 (24–39)

Data are presented as the medians (25th–75th percentile); TSAT, transferrin saturation; DM, diabetes mellitus; IGGT, impaired gestational glucose tolerance; ALAT, alanine aminotransferase; ASPAT, aspartate aminotransferase.

**Figure 7:**
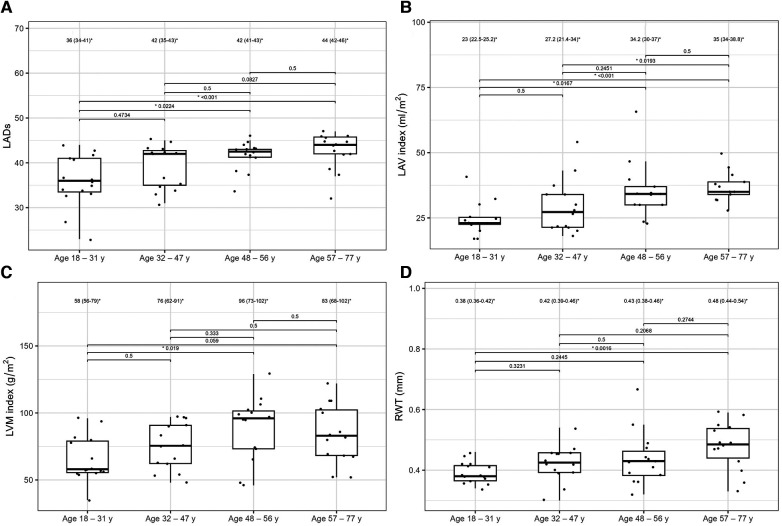
LADs (**A**), LAV index (**B**), LVM index (**C**), and RWT (**D**) values between the HH patients stratified by their age. The dots show the values obtained by each person analyzed. The values above the brackets correspond to the *p*-value. * Data are presented as medians (25th–75th percentile).

**Figure 8:**
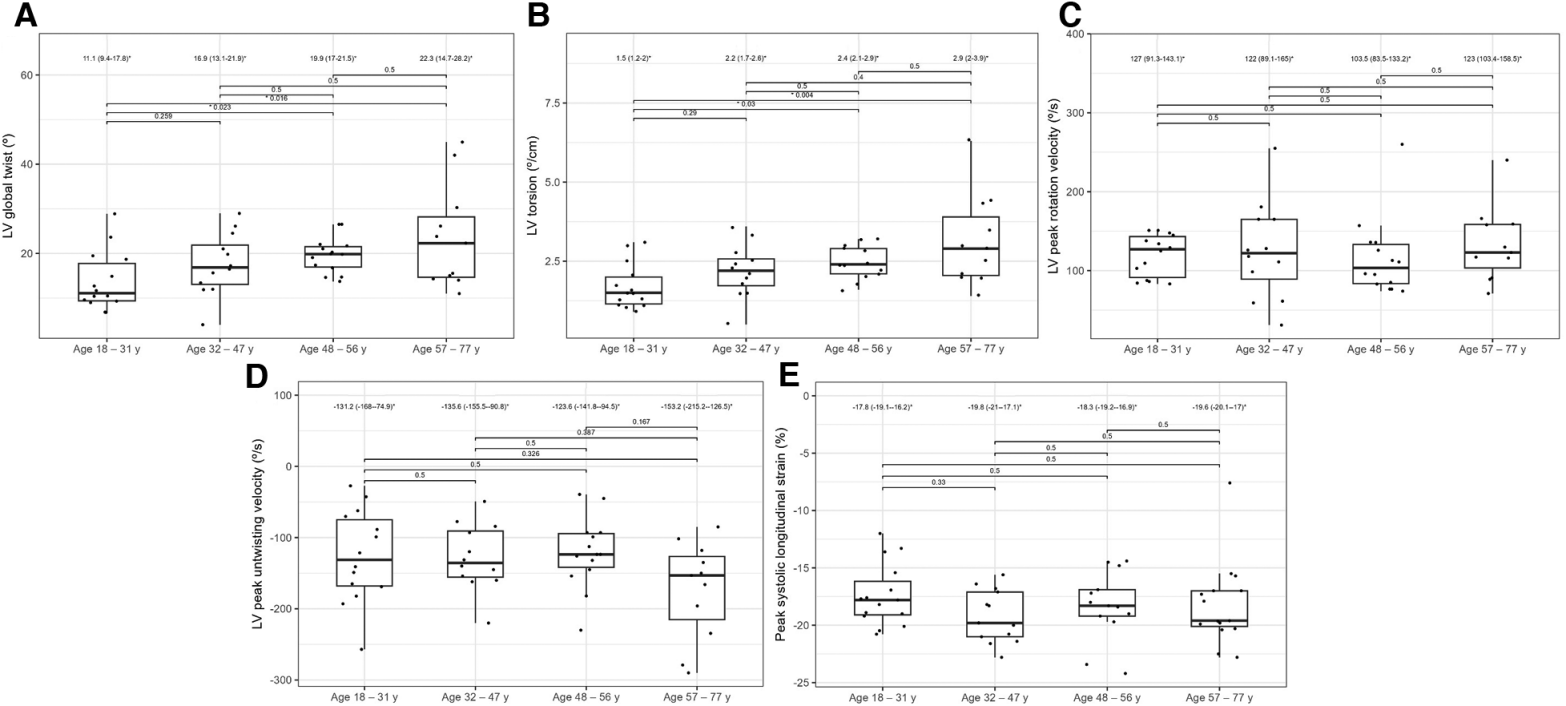
Graphs showing comparisons of LV global twist (**A**), LV torsion (**B**), LV peak rotation velocity (**C**), LV peak untwisting velocity (**D**), and peak systolic longitudinal strain (**E**) values between the HH patients stratified by their age. The dots show the values obtained by each person analyzed. The values above the brackets correspond to the *p*-value. * Data are presented as medians (25th–75th percentile).

Table 6 compares the standard and 2D STE echocardiographic parameters of patients with HH stratified by age. Some standard parameters related to diastolic function (LA size, IVS, RWT, LVM index, Em, and E/Em) changed above the normal range most prominently in the third decade of life (Figure 7). In contrast, some STE parameters related to systolic function (LV global twist and torsion) increased significantly from the fourth decade of life (Figure 8).

**Table 6:** Comparison of standard and 2D STE echocardiographic parameters between the HH patients stratified by their age.

	Minimum to Q25 Age 18–31 year *n* = 17	Q25–Q50 Age 32–47 year *n* = 14	Q50–Q75 Age 48–56 year *n* = 13	O75 to maximum Age 57–77 year *n* = 14	*p*
IVS (mm)	9 (8–10)^§,¶^,^	11.0 (10.0–12.8)*	11 (10–12)*	11 (10–12)*	0.008
PW (mm)	8 (7–9)^¶^^,^^	10 (9–11)	10 (9–11)*	10 (9–11)*	0.012
Em (cm/s)	0.14 (0.13–0.16)^¶^^,^^	0.11 (0.09–0.13)^	0.09 (0.08–0.1)*	0.08 (0.07–0.09)*^,^^§^	<0.001
E/Em	5.6 (4.8–6.7)^¶^^,^^	6.4 (5.6–7.8)^	7.8 (7.5–9.2)*	9 (7–10)*^,^^§^	<0.001
LVEDD (mm)	47 (43–48)	47 (45–48)	47 (45–51)	43 (42–45)	0.14
LVESD (mm)	28 (26–31)	28 (27–31)	29 (25–30)	25 (24–28)	0.398
LVEF (%)	57 (53–62)	58 (54–61)	60 (59–63)	61 (60–66)	0.111
RVID (ms)	26 (23–28)	28.5 (25–30)	27 (26–30)	27 (25–28)	0.129
LV basal rotation (°)	−4.2 (−5.7–−1.7)	−3.9 (−6.3–−2.6)	−7.6 (−11.0–−4.0)	−6.0 (−7.0–−4.2)	0.089
LV basal rotation velocity (°/s)	−50.5 (−63.6–−0.0)	−55.1 (−75.2–−17.3)	−49.7 (−75.0–−32.3)	−63.1 (−80.9–−42.8)	0.762
LV basal untwisting velocity (°/s)	44.7 (15.2–67.8)	45.0 (34.1–51.0)	77.81 (40.0–80.1)	66.0 (39.0–78.2)	0.188
LV apical rotation (°)	7.8 (5.3–15.1)	12.6 (10.2–16.7)	13.0 (9.0–18.0)	13.2 (11.0–22.9)	0.098
LV apical rotation velocity (°/s)	67.0 (37.2–101.3)	98.1 (63.4–119.6)	82.0 (51.9–87.0)	89.8 (66.0–129.5)	0.227
LV apical untwisting velocity (°/s)	−82.8 (−105.4–−64.2)	−75.7 (−116.1–−60.2)	−76.0 (−119.6–−55.1)	−107.8 (−156.3–−93.9)	0.335

Data are presented as medians (25th–75th percentile). IVS, intraventricular septum (mm); PW, posterior wall; E, early mitral velocity; Em, peak mitral annulus velocity; E/Em, early mitral inflow velocity to peak mitral annulus velocity ratio; LVEDD, left ventricle end-diastolic diameter; LVESD, left ventricle end-systolic diameter; LVEF, left ventricular ejection fraction; RVID, right ventricle internal diameter; LV, left ventricle. *p*-value: for differences among all groups with Kruskal–Wallis test for continuous variables or with chi-square test for categorical variables, *p* < 0.05 in posthoc tests for differences with group age 18–31 year (*), age 32–47 year (^§^), age 48–56 year (^¶^) or age 57–77 year (^).

### 3.4. The correlations between iron turnover parameters and echocardiographic parameters

Table 7 presents the correlations between iron turnover parameters and measured echocardiographic parameters. Some correlations were observed between the time from diagnosis and the number of venesections with echocardiographic parameters. The correlations were significant; however, they were relatively weak (the *r*-values were lower than 0.7).

**Table 7:** Correlations between iron turnover, time from diagnosis, number of venesections and 2D STE parameters in HH patients.

Echocardiographic parameter	Iron		Ferritin		TSAT		Time from diagnosis		Numbers of venesections	
	*r*	*p*	*r*	*p*	*r*	*p*	*r*	*p*	*r*	*p*
LADs	0.11	0.414	0.058	0.669	0.161	0.239	0.189	0.16	0.164	0.224
LAA index (cm^2^/m^2^)	−0.001	0.996	0.032	0.820	−0.025	0.863	0.295	0.032	0.336	0.014
LAV index (ml/m^2^)	0.119	0.39	0.074	0.594	0.050	0.720	0.395	0.003	0.408	0.002
IVS (mm)	0.035	0.797	0.278	0.036	−0.009	0.949	0.522	0.001	0.342	0.009
PW (mm)	0.212	0,114	0.282	0.033	0.007	0.96	0.496	0.001	0.419	0.001
RWT	0.200	0.136	0.244	0.067	0.004	0.975	0.259	0.051	0.247	0.064
LVM index (g/m^2^)	0.117	0.385	0.303	0.022	0.087	0.522	0.530	0.001	0.433	0.001
Em	−0.005	0.971	−0.101	0.459	−0.147	0.284	−0.414	0.001	−0.399	0.002
E/Em	0.082	0.548	0.082	0.548	0.141	0.303	0.340	0.01	0.394	0.002
LVEDD (mm)	0.095	0.504	0.172	0.200	0.112	0.409	0.195	0.145	0.156	0.247
LVESD (mm)	0.090	0.481	−0.051	0.706	−0.004	0.977	0.211	0.115	0.084	0.535
LVEF (%)	0.110	0.451	0.112	0.442	0.002	0.987	0.219	0.130	0.107	0.463
RVID (ms)	−0.002	0.987	0.346	0.008	0.098	0.472	0.262	0.049	0.254	0.056
LV basal rotation (°)	0.226	0.107	0.035	0.81	0.129	0.365	−0.302	0.03	−0.21	0.135
LV basal rotation velocity (°/s)	0.14	0.324	−0.057	0.689	0.094	0.514	−0.279	0.045	−0.2	0.156
LV basal untwisting velocity (°/s)	−0.229	0.102	−0.183	0.200	−0.173	0.225	0.343	0.013	0.256	0.067
LV apical rotation (°)	−0.055	0.704	0.104	0.473	−0.095	0.512	0.238	0.092	0.266	0.059
LV apical rotation velocity (°/s)	−0.007	0.962	−0.077	0.595	−0.037	0.800	0.211	0.137	0.161	0.26
LV apical untwisting velocity (°/s)	0.071	0.621	0.098	0.500	0.122	0.4	−0.144	0.315	−0.29	0.84
LV global twist (°)	−0.145	0.310	0.083	0.569	−0.125	0.387	0.342	0.014	0.337	0.015
LV torsion (°/cm)	−0.13	0.366	0.096	0.514	−0.053	0.717	0.316	0.025	0.35	0.013
LV peak rotation velocity (°/s)	−0.186	0.192	−0.061	0.675	−0.089	0.539	0.131	0.361	0.018	0.899
LV peak untwisting velocity (°/s)	0.113	0.429	0.236	0.099	0.156	0.28	−0.238	0.093	−0.055	0.704
Peak systolic longitudinal strain (%)	−0.026	0.848	−0.016	0.907	0.061	0.659	0.261	0.052	0.154	0.256

LADs, left atrial diameter; LAA index, left atrium area/m^2^; LAV index, left atrium volume/m^2^; IVS, intraventricular septum (mm); PW, posterior wall; RWT, relative wall thickness; LVM index, left ventricle mass/m^2^; E, early mitral velocity; Em, peak mitral annulus velocity; E/Em, early mitral inflow velocity to peak mitral annulus velocity ratio; LVEDD, left ventricle end-diastolic diameter; LVESD, left ventricle end-systolic diameter; LVEF, left ventricular ejection fraction; RVID, right ventricle internal diameter; LV, left ventricle.

## 4. Discussion

Our results support the role of echocardiography in revealing the differences between patients with HH in terms of the time from the initial diagnosis and the patient age. To the best of our knowledge, this is the first study to address this issue in patients with HH.

### 4.1. The potential impact of HH on the heart considering the stage of the disease

Standard echocardiography revealed differences in diastolic parameters in the enrolled patients with HH compared with healthy controls. Several authors have documented diastolic dysfunction in patients with HH (14–16). The cited studies' techniques were based on the standard two-dimensional echocardiography, including M-mode (16) and TDI techniques (14, 15). For example, Candell-Riera et al., showed that patients with idiopathic hemochromatosis presented significantly higher LADs index (22.2 ± 3.7 mm/m^2^) and LVM index (150 ± 56.2 g/m^2^) compared to the control group (respectively 19.2 ± 1.7 mm/m^2^ and 91.7 ± 14.6 g/m^2^, respectively) (16). Similarly, Palka et al. reveled deviations in the LADs and the LVM index in their analysis of patients with HH, with 83% being C282Y homozygotes (15). The authors also observed significantly higher values of LVM index (101 ± 26 g/m^2^) and LADs in patients with HH (3.81 ± 0.74 cm) compared to healthy controls (77 ± 12 g/m^2^ for LVM index and 3.27 ± 0.39 cm for LADs, respectively) (15). Our results are in line with this study: we found significantly higher LADs [42.0 (36.0–43.0) mm/m^2^] and LAV index values [31.0 (23.0–36.5) ml/m^2^], and LVM index values [78.0 (58.0–96.0) g/m^2^] in patients with HH compared to healthy volunteers (37.0 [34.0–39.0] mm/m2 for LADs, 21.5 [19.0–27.1] ml/m2 for LAV index, and 66.0 [53.0–72.0] g/m2 for LVM index, respectively) (Figure 1). Interestingly, Davidsen et al. did not show such differences in the LADs and LVM index values between patients with HH and healthy controls, possibly because all of their patients were regularly treated with venesections (14).

Furthermore, we compared patients with HH at the different stages of the disease. To the best of our knowledge, this is the first such analysis of patients with HH. We revealed worse diastolic parameters measured by standard echocardiography in patients with HH compared to controls, with the most noticeable differences (in morphological and functional parameters) detected between the healthy and long-lasting HH group. The latter had worse LV wall thickness, RWT, LVM index, and Em indices, probably due to the disease's length and the patient's age (Table 4, Figure 3). Hypertension and glucose disturbances, which occur earlier in patients with HH than in the overall population, could have an additional impact on the left ventricular diastolic function (17).

The application of 2D STE in our study allowed for a more precise description of systolic dysfunction. In one of our previous studies, the patients with recently diagnosed HH had worse rotation and strain parameters measured by 2D STE than healthy controls, despite the lack of differences in standard echocardiographic parameters (Figure 4) (10). We confirmed this observation in the presented study of patients with HH at the different stages of the disease.

2D STE may allow for the complex analysis of heart mechanics. A decrease in the untwisting velocity represents a predominance of diastolic dysfunction, and significant decreases in rotation, rotation rate, twist, torsion, and peak systolic longitudinal strain are critical predictors of systolic dysfunction (18–20). Only a few studies (10, 11, 21–23) have used speckle-tracking analysis in the context of iron-overload symptoms. However, these studies involved patients with beta-thalassemia major, an extreme model of systemic iron overload (24). In our study, the recently diagnosed HH group was characterized by the worst parameters of the LV rotation, the twisting and untwisting indices, whereas the percentages of hypertension and glucose disturbances were the lowest in that group compared to the other HH groups (Table 4; Figure 4), which may exclude the possible influence of these comorbidities on 2D STE parameters. The aforementioned rotation indices improved significantly in the medium and long-lasting groups of patients, possibly due to HH treatment (Table 4; Figure 4). This postulation aligns with previous data in the literature (11, 25). For example, Byrne et al. showed that intensification of venesection therapy may significantly improve radial strains following a 1-year course of phlebotomies (11). In one of our clinical case presentations, we observed similar changes in the rotation, twist, and torsion LV parameters after 6-month therapy with venesections in our patient with recently diagnosed HH (25). However, we did not inestigate the direct influence of treatment but compared the patients at the different HH stages. Our results make it unclear why the Long-lasting HH patients did not present further improvement in rotation parameters. That could be explained in two ways: the initial treatment by venesections could only normalize rotation parameters in HH patients without further improvement, and the aging of the enrolled patients could implicate our results (26). However, these explanations must be verified in a further prospective study with the appropriate follow-up observations.

The peak systolic longitudinal strain values in this study were significantly lower in patients with HH at all stages of the disease (Figure 4). This parameter did not improve with treatment initiation and continuation, which suggests early irreversible changes in the hearts of patients with HH starting at the early stages of the disease. We obtained similar results in our previously published case report, where peak systolic longitudinal strain did not improve within the treatment (25). This finding may have a substantial clinical impact since an impaired peak longitudinal strain value is a well-established prognostic factor for cardiac complications (27–29). However, further research is necessary to verify whether the differences in the peak systolic longitudinal strain between the healthy volunteers and patients with HH at different disease stages are clinically relevant. The LVEF was within the normal range in our patients, despite statistically significant differences between the patients with HH and controls. This finding is consistent with previously documented data (16), indicating that LVEF may not be the best parameter for assessing systolic damages in the patients with HH.

### 4.2. The potential impact of HH on the heart considering the patient's age

Iron overload may accelerate the aging of the cardiovascular system, including the heart, through oxidative stress, which results in appropriate changes in diastolic and systolic parameters. These changes can be detected by standard echocardiography (increase in the LV width and LVM index, LA enlargement, or changes in mitral flow parameters) or by STE (decrease in rotation, twist, and torsion values of the LV) (10, 15–17).

In the presented study, the echocardiographic features of myocardial walls' thickening in patients with HH appeared more rapidly than in healthy idividuals. For instance, Ganau et al. evaluated the effect of age on concentric remodeling in healthy participants and showed that RWT values increased with age and constituted 0.32 ± 0.044 among patients younger than < 41 years, 0.34 ± 0.05 for those aged 41–64 years, and 0.37 ± 0.050 for those > 64 years (30). Our patients with HH achieved similar RWT values faster than healthy individuals in the cited study (Figure 7). In our study, the LVM index was 76 (62–91) g/m^2^ in the fourth decade, whereas Kaku et al. reported a lower maximum value of the LVM index in the older (the eighth decade of life) healthy volunteers (31). Another important parameter that increases with age is the LA size. Nikitin et al. showed that the LAD was 3.51 ± 0.51 cm in patients aged 20–39 years, 3.99 ± 0.54 cm in those aged 40–59 years, 4.12 ± 0.43 cm in those aged 60–79 years, and 4.19 ± 0.51 in those aged > 80 years (32). In our analysis, the LADs increased slightly faster (Figure 7). Similarly, the LAV index increased faster in our patients (Figure 7) than in healthy individuals, as reported by Nikitin et al. (32). Furthermore, we showed increased E/Em values in the older patients compared to the younger patients; however, we did not find any significant differences from the general population (31).

Regarding LVEF, as an index of systolic function, Kaku et al. showed that it does not significantly change with age (31). Similarly, we did not observe any changes in LVEF in our patients with HH with age; however, LVEF was slightly lower than that of the general population (31). STE may allow for a better understanding of age-related systolic function changes than a standard echocardiography with LVEF measurments. According to Kuznetsova et al., the absolute values of the peak systolic longitudinal strain significantly decreased in a healthy population with age: 24.2 ± 3.4%, in individuals aged < 40 years, 23.5 ± 3.19% in those aged 40–59, and 22.3 ± 3.62% in those aged > 60 years (33). Among our patients with HH, peak systolic longitudinal strain was at a lower level: −17.8 (−19.1–16.2) % in patients with HH aged 18–31 years, and was without significant changes in the older patients (among patients aged 32–47: −19.8 [−21.0–17.1] %, aged 48–56: 18.3 [−19.2–16.9] %, aged 57–77: 19.6 [−20.1–17.0]%; Figure 8). Kaku et al. reported that the LV global twist and torsion values increased with age (34). According to that study, the maximum LV twist and torsion values were reached in the seventh decade of life (12.7 ± 4.8° for LV twist and 1.75 ± 0.66°/cm for torsion). In our analysis, similar LV twist values were present in patients with HH at a younger age (18–31 years old) and constituted 11.1 (9.4–17.8)° for twist and 1.5 (1.2–2.0)°/cm for torsion (Figure 8). Increasing the rotation parameters within the age range may result in a decrease in the longitudinal strain of the LV.

Our results showed that thickening of the myocardium and dilatation of the LA occurred faster in patients with HH than in the general population. Similarly, the deterioration in systolic function detected using STE was more prominent in patients with HH than in the general population. This could suggest a harmful effect of the disease on the heart in addition to the aging of the patients; however, this hypothesis needs to be verified.

### 4.3. Correlation of the obtained results with the parameters of iron metabolism

The presented analysis showed that the correlations between the iron turnover parameters and echocardiographic indices were relatively weak. These findings align with data from the literature (23, 35, 36) and our previous results, including cardiac magnetic resonance assessments (10, 37). There is probably no “direct” relationship between the levels of iron turnover parameters and myocardial function, and myocardial iron overload is not the only mechanism involved in the development of HH cardiomyopathy (1). The precise mechanism of heart involvement in HH is yet to be entirely understood. The excessive capacity of serum transferrin to bind iron, which is typical in patients with HH, results in uncontrolled iron entry into cardiomyocytes, which may increase their susceptibility to oxidative cell stress (3). Cardiomyocytes contain many mitochondria, causing the myocardium to be more vulnerable to oxidative stress damage. Free iron ions that damage the mitochondrial and nuclear DNA may activate fibroblast proliferation and differentiation into myofibroblasts, which are responsible for heart fibrosis (38). Oxidative stress results in the peroxidation of cell membranes, particularly those in the mitochondria, which reduces the amount of adenosine triphosphate generated during oxidative phosphorylation. Oxidative stress impairs heart muscle relaxation and delays contraction by inhibiting SERCA2 enzyme activity and increasing the cytoplasmic concentration of calcium ions in cardiomyocytes. Therefore, amlodipine, a Ca channel blocker, may be promising as an agent to potentially reduce oxidative stress during iron overload (39).

Magnetic resonance imaging is the gold standard for measuring iron overload (2). Previous studies presented changes in the heart documented on cardiac magnetic resonance (CMR) in patients with beta-thalassemia, most of whom met the current criteria for iron overload in terms of myocardial T2 * < 20 ms (40–42). HH is not the same iron overload model as beta-thalassemia, which has been documented in our previous study (37). In all the studied patients with HH, myocardial T2*, T1, and T2* did not fulfill the clinically severe iron overload (20 ms) threshold, which confirms the hypothesis that in modern patients with HH not simple iron storage in the heart could play an essential role in the heart damage process. Therefore, in the present study, we assessed the role of echocardiography, particularly STE, in identifying differences in patients with HH at various disease stages and ages, rather than calculating iron overload.

## 5. Clinical implications

HH involves a chronic process of complicated iron dysregulation cycles with unavoidable cardiac involvement, leading to changes in the diastolic and systolic functions of the heart. In addition to genetic testing and iron parameter measurement, detecting cardiac involvement early, even in asymptomatic individuals, and utilizing contemporary imaging techniques may have tremendous clinical utility, particularly for monitoring the course of the disease and possible aging of the heart from the oxidative stress generated by free iron ions in patients with HH. Differences in the diastolic parameters can be easily detected using standard echocardiography. In contrast, advanced echocardiography using 2D STE is necessary to better reveal systolic changes that appear notably attenuated and irreversible in patients with HH at different stages of the disease.

## 6. Study limitations

Our study had some critical limitations. The first was the relatively small sample size, which resulted from the highly selective enrollment process (patients without any cardiovascular symptoms), the demand to achieve the best image quality (frame rate of 50–80 frames/s), and the inclusion of only patients with genetically confirmed HH and without cardiological comorbidities (apart from hypertension). We presented the data of different patients, rather than evaluating the same patients over the years of venesections. We did not present a CMR assessment and could not address the iron overload status. This was a pilot study, and further studies with more homogeneous patients are required.

## 7. Conclusions

Echocardiography can reveal possible heart damage in patients with HH at different disease stages and highlight features of accelerated myocardial aging in these patients. Further studies with follow-up observations are required to provide a better understanding of these issues.

## Data Availability

The raw data supporting the conclusions of this article will be made available by the authors, without undue reservation.
